# Deciphering the Role of Holin in Mycobacteriophage D29 Physiology

**DOI:** 10.3389/fmicb.2020.00883

**Published:** 2020-05-08

**Authors:** Varun Rakeshbhai Bavda, Vikas Jain

**Affiliations:** Microbiology and Molecular Biology Laboratory, Department of Biological Sciences, Indian Institute of Science Education and Research (IISER), Bhopal, India

**Keywords:** phage infection, phage therapy, mycobacteria, transmission electron microscopy, recombineering

## Abstract

In the era of antibiotic resistance, phage therapy is gaining attention for the treatment of pathogenic organisms such as *Mycobacterium tuberculosis*. The selection of phages for therapeutic purposes depends upon several factors such as the host range that a phage can infect, which can be narrow or broad, time required for the host cell lysis, and the burst size. Mycobacteriophage D29 is a virulent phage that has the ability to infect and kill several slow- and fast-growing mycobacterial species including the pathogenic *M. tuberculosis*. It, therefore, has the potential to be used in phage therapy against *M. tuberculosis*. D29 lytic cassette encodes three proteins *viz*. peptidoglycan hydrolase (LysA), mycolylarabinogalactan esterase (LysB), and holin, which together ensure host cell lysis in a timely manner. In this work, we have scrutinized the importance of holin in mycobacteriophage D29 physiology. Bacteriophage Recombineering of Electroporated DNA (BRED) approach was used to generate D29 holin knockout (D29Δ*gp11*), which was further confirmed by the Deletion amplification detection assay (DADA)-PCR. Our results show that D29Δ*gp11* is viable and retains plaque-forming ability, although with reduced plaque size. Additionally, the host cell lysis governed by the mutant phage is significantly delayed as compared to the wild-type D29. In the absence of holin, D29 shows increased latent period and reduced burst size. Thus, our experiments show that while holin is dispensable for phage viability, it is essential for the optimal phage-mediated host cell lysis and phage propagation, which further points to the significance of the “clock” function of holin. Taken together, we show the importance of holin in governing timely and efficient host cell lysis for efficient progeny phage release, which further dictates its critical role in phage biology.

## Introduction

Tuberculosis (TB) is an infectious disease caused by the pathogenic *Mycobacterium tuberculosis*. With an estimated death of 1.5 million people in year 2018, TB is among top 10 cause of death (Harding, 2020). Further in recent years, TB epidemic has been enhanced by the rapid emergence of multi drug resistant and extensively drug resistant *M. tuberculosis* strains (Koul et al., 2011; Coll et al., 2018). Thus being a global health threat, TB requires an urgent need of alternative approach to combat the failure of treatment with antibiotics.

Mycobacteriophages are viruses that require mycobacterial host for their propagation (Sarkis and Hatfull, 1998; Hatfull, 2014a, b, 2018). Being the natural killer of their hosts, mycobacteriophages have the potential to be developed as the next-generation phage-based therapeutics against TB and other pathogenic mycobacterial infections, especially when the antibiotics against the pathogens become ineffective. Indeed, mycobacteriophages have not only shown their potential to treat *Mycobacterium abscessus* infection (Dedrick et al., 2019), but they have also been used for the development of phage-based diagnostics (Jacobs et al., 1993; Pearson et al., 1996; Park et al., 2003). Among the many mycobacteriophages, D29, which is a virulent phage and is capable of infecting and killing both slow and fast growing mycobacterial species (Ford et al., 1998; Rybniker et al., 2006), and its lysis enzymes have been used to kill *M. tuberculosis* and other mycobacteria in various animal-based experiments (Carrigy et al., 2019; Fraga et al., 2019). The host cell lysis is governed by the protein products of the “Lytic cassette” genes. In D29, the lytic cassette comprises *gp10* coding for an endolysin LysA that targets the peptidoglycan cell wall and *gp12* producing LysB that targets the outer mycolic acid layer of mycobacteria (Payne et al., 2009; Fraga et al., 2019). Additionally, *gp11*, which is sandwiched between *gp10* and *gp12*, produces holin that perforates the cell membrane of mycobacteria thus allowing for the diffusion of the hydrolytic enzymes to the periplasm and ensuing cell lysis (Catalao et al., 2013; Kamilla and Jain, 2016).

Data available from other phages suggest that holin not only allows for the lysins release to the periplasm, but it also functions as a “clock”, and times the lysis event during phage infection (Wang et al., 2000; Grundling et al., 2001). Holins that form large holes to allow the diffusion of folded lysins to the periplasm are called canonical holin (Wang et al., 2003; Savva et al., 2014; To and Young, 2014). On the other hand, several phages code for pinholins that form small holes that disrupt proton gradient across the membrane, but are not involved in the periplasmic delivery of endolysins (Park et al., 2007; Catalao et al., 2010; Young, 2013; Cahill and Young, 2019). Holin, therefore, times the lysis of the host cell and hence functions as “clock” (Wang et al., 2000). In the case of D29, although the holin protein has been shown to form large holes (Catalao et al., 2011; Kamilla and Jain, 2016), its endolysin LysA, however, can translocate to periplasm even in the absence of holin when it is expressed in *Mycobacterium smegmatis* (Pohane et al., 2014).

The functioning of λ phage holin has been dissected in great detail. It has been suggested that holin, upon its expression, initially accumulates in the plasma membrane without disrupting the membrane integrity. This gives sufficient time for the progeny phages to assemble inside the host cell. After reaching a threshold concentration in the membrane, holin spontaneously changes its structure and forms mature holes (Ryan and Rutenberg, 2007; White et al., 2011). These holes dissipate the proton motive force across the plasma membrane and allow the free diffusion of endolysins already accumulated within cytoplasm (Wang et al., 2000; Grundling et al., 2001; Saier and Reddy, 2015). It is conceivable, therefore, that by controlling the timing of lysis, holin determines the duration of the infective cycle of phage, and that an alteration in this function may lead to premature or delayed host lysis, resulting in a loss of phage fitness either in terms of progeny phage number or loss of progeny phages, respectively (Wang, 2006).

While holins from other phages have been studied in great detail (Wang et al., 2000), the structure-function relationship of mycobacteriophage holin and how it regulates mycobacterial cell lysis are not well characterized. We have previously shown that D29 holin possesses two transmembrane domains (TMD) at its N-terminus and a highly charged C-terminal region (Kamilla and Jain, 2016; Lella et al., 2016), and that its expression is extremely toxic to both *Escherichia coli* and mycobacteria. However, an observation that the expression of D29 LysA in *M. smegmatis* is sufficient to kill the bacterium even in the absence of holin (Pohane et al., 2014) tempted us to speculate if holin is an essential protein for the D29 phage survival. Therefore, in order to understand the role of holin in phage biology, we deleted holin gene from D29 phage genome and examined its physiology. Here we show that D29 phage devoid of holin, although viable with poor plaque forming ability, shows delayed host cell lysis, which may hint towards the importance of “clock” function of this protein in mycobacteriophage biology.

## Materials and Methods

### Bacterial Strains, Plasmid, Media, and Growth Conditions

Wild-type *M. smegmatis* strain mc^2^155 was grown at 37°C with constant shaking unless specified otherwise in Middlebrook 7H9 (MB7H9) (Difco) medium supplemented with 2% glucose and 0.05% tween 80. For recombineering experiments, electrocompetent *M. smegmatis* cells carrying pJV53 plasmid were prepared as described previously (van Kessel and Hatfull, 2007; Marinelli et al., 2008). For all phage infection experiments, *M. smegmatis* was grown in MB7H9 supplemented with 10% OADC (Difco) along with 1 mM CaCl_2_. For preparation of solid culture medium, 1.5% agar and 2% glucose were additionally added to the growth medium. For soft agar preparation, 0.75% agar was added to the growth medium in order to perform agar overlay for phage infection experiments. In all experiments, cultures were incubated at 37°C.

### Deletion of Holin Gene in D29 Phage and Its Confirmation

Bacteriophage Recombineering of Electroporated DNA (BRED) assay was performed to generate holin knockout of D29 phage essentially as described previously (van Kessel and Hatfull, 2007; Marinelli et al., 2008). Briefly, the allelic exchange substrate (AES) was generated by PCR using the primers listed in Table 1, confirmed by DNA sequencing, and co-electroporated with D29 genomic DNA in *M. smegmatis* carrying pJV53 plasmid. Screening of plaques was carried out by Deletion Amplification Detection Assay (DADA) PCR (Swaminathan et al., 2001; Marinelli et al., 2008) using the primers P5 and P6 as listed in Table 1. The plaques were further purified to obtain pure mutant phage essentially as described previously (Joshi et al., 2019). Briefly, primary plaques which showed DADA PCR amplification corresponding to both wildtype and knockout phages were selected and diluted further for carrying out phage infection with *M. smegmatis* cells to isolate pure holin knockout phage. The pure holin knockout phage (D29Δ*gp11*) was further confirmed by DNA sequencing. Pairwise alignment of the sequencing data with the predicted genomic DNA sequence was carried out using CLUSTAL Omega (Madeira et al., 2019).

**TABLE 1:** List of primers used in the present study.

S. No.	Primer name	Primer sequence (5′–3′)
1	P1	GCTCCCCCGAGTTCATCGCAC
2	P2	CCAGGGCTTGCTCATAGGGCTCCATTCCTGG
3	P3	GAGCCCTATGAGCAAGCCCTGGCTGTTCACC
4	P4	GATGTCAAGTACGTCGCGTGCCGTATCG
5	P5	CCAGAGGATCCGGCGACCAACTACTGACTC
6	P6	GAACGGTGAACAGCCAGGGCTTGCTC

### Western Blotting Analysis

*M. smegmatis* cells were grown at 37°C till OD_600_ of the culture reached 0.6. Bacteria were infected with either D29WT or D29Δ*gp11* at multiplicity of infection (MOI) of 1 for 1 h at 37°C. Cells were then harvested and resuspended in a lysis buffer containing 40 mM Tris-Cl pH 8.0, 200 mM NaCl, 5 mM imidazole, 5 mM 2-mercaptoethanol and 8 M urea, and lysed by sonication. Cell lysate thus obtained was centrifuged at high speed and the supernatant was mixed with SDS-loading dye to separate the proteins on polyacrylamide gel. The production of LysA and LysB proteins was examined by western blotting essentially as described previously (Pohane et al., 2014) using anti-LysA (Pohane et al., 2014) and anti-LysB antibodies. Polyclonal anti-LysB antibodies were raised in rabbit using purified LysB protein (Bioneeds India Private Limited, India).

### Host Cell Viability and Cell Lysis Attributed by Phage

*M. smegmatis* log phase culture was infected with either D29WT or D29Δ*gp11* phage at MOI of 1, and the viability of cells after phage infection was measured using AlamarBlue (BIO-RAD) cell viability reagent essentially as described previously (Warrier et al., 2012; Joshi et al., 2019; Patil and Jain, 2019) at different time points post-infection. Phage-induced bacterial cell lysis was also monitored by measuring the optical density of the culture at 600 nm (OD_600_) at specified time points on a spectrophotometer (Jenway, Bibby scientific). The amount of ATP released into the culture supernatant upon cell lysis was determined by BacTiter-Glo Microbial Cell Viability Assay kit (Promega) as described previously (Joshi et al., 2019; Patil and Jain, 2019).

### Transmission Electron Microscopy (TEM) Imaging of Phages

Imaging of phages on a transmission electron microscope was carried out as described previously (Ackermann, 2009). Briefly, both D29WT and D29Δ*gp11* were purified in high titre with at least 1 × 10^11^ PFU/ml. Formvar/Carbon coated Cu grid (400 mesh) was procured from TED-PELLA, INC. Approximately 5–10 μl of filtered phage was put onto the Cu grid and after a brief incubation (5–10 min) at room temperature, the extra liquid was removed by blotting paper. A drop of 2% uranyl acetate stain was applied on the grid and was incubated for not more than 1 min. Extra stain was then removed by blotting paper, and the grid was washed thrice with Milli-Q water. The grid was then allowed to dry in a desiccator overnight at RT. Grid was then used for TEM imaging on FEI Talos 200S system equipped with a 200-kV field emission gun.

### Phage Adsorption Kinetics and One Step Growth

Mycobacteriophage adsorption kinetics was measured as described previously (Delbruck, 1940; Hendrix and Duda, 1992; Kropinski, 2009), with some modifications. Briefly, 1 ml of log phase culture (OD_600_ ∼0.6) of *M. smegmatis* was mixed with either D29WT or D29Δ*gp11* keeping MOI of 1. At regular intervals, equal amount of phage-infected culture was collected in each case and immediately centrifuged at 14000 rpm at 4°C to separate uninfected and infected host from free phages. The number of free phages present in the supernatant thus obtained was estimated by performing phage infection and plotted as percentage of free phages with respect to time 0 being 100% (no infection). One step growth was performed for both D29WT and D29Δ*gp11* essentially as described previously (Hyman and Abedon, 2009; Catalao et al., 2011). Briefly, at regular time intervals, 1 ml of diluted phage-infected sample was used to estimate number of phage particles present in it by carrying out secondary infection. Relative phage titre was determined and was plotted (Hyman and Abedon, 2009).

## Results

### Construction of Mycobacteriophage D29 Holin Knockout

**FIGURE 1:**
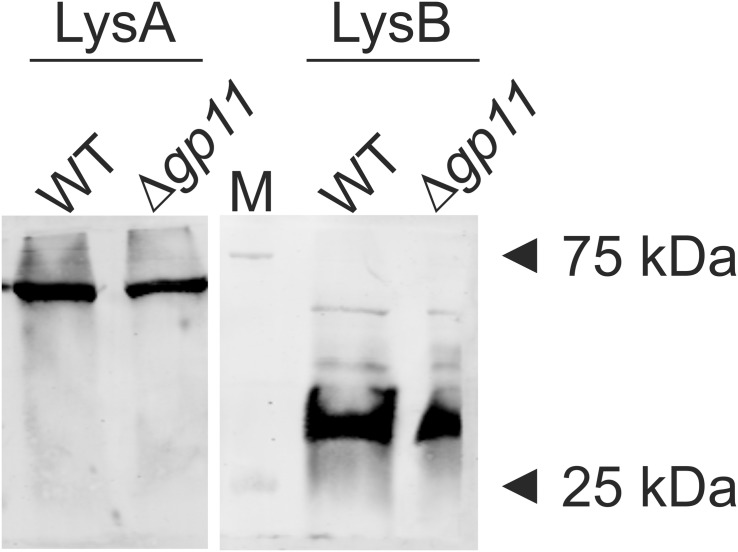
LysA and LysB protein expression analysis by western blotting. The blots show the production of LysA and LysB by both D29WT (WT) and holin knockout (Δ*gp11*) phages. Only one representative blot in each case is shown here. “M” depicts molecular weight marker with two bands marked.

D29 lytic cassette possesses three overlapping genes *viz*. *gp10*, *gp11*, and *gp12*. In order to generate a holin knock-out (D29Δ*gp11*), we followed the BRED strategy (Marinelli et al., 2008), which we used earlier to also mutate the gp10 gene in D29 (Joshi et al., 2019). The schematic of the holin deletion is presented in Supplementary Figure S1A. The 100 bp upstream and 99 bp downstream regions of *gp11* were PCR amplified and fused by overlapping PCR to generate AES of 199 bp (Supplementary Figures S1B,C). It is important to note here that the start codon of *gp11* is overlapped with the stop codon of *gp10*; similarly, the stop codon of *gp11* overlaps with the start codon of *gp12* (Supplementary Figure S2). Therefore, the overlapping regions were retained in this fashion in the AES so that *gp10* and *gp12* share the same overlapping region and the open reading frame is not changed. Next, to generate D29Δ*gp11*, purified genomic DNA of D29 and AES were co-electroporated into electrocompetent *M. smegmatis* cells containing pJV53 plasmid as shown in schematic (Supplementary Figure S1D). The pJV53 encodes for recombinase that allows for the recombination to occur between the AES and its homologous region of the phage genomic DNA (Marinelli et al., 2008). The recovered cells were plated using soft agar method and the plaques thus obtained (Supplementary Figure S3A) were screened for the mutated phage by DADA PCR (Marinelli et al., 2008). Initially-obtained plaques were found to be having both the knock-out and the wild-type (WT) phages (Supplementary Figure S3B). Secondary and further phage infections as shown in the schematic (Supplementary Figure S3C) were then carried out to obtain pure D29Δ*gp11* phage, which gave correct size amplicon of ∼400 bp in PCR (Supplementary Figure S3D); the amplicon was further sequenced to confirm the deletion of *gp11* (Supplementary Figure S4). We next performed western blotting of the D29WT- and D29Δ*gp11*-infected *M. smegmatis* culture to assess the expression of *gp10* and *gp12* genes during phage infection. We found both of these proteins being produced during infection (Figure 1), which indicates that the deletion of holin does not hamper expression of the neighboring genes.

### Deletion of Holin Results in Smaller Plaques by D29 Phage

**FIGURE 2:**
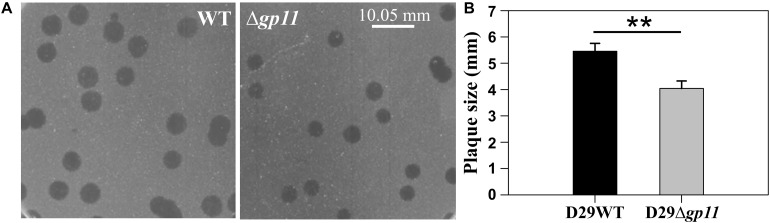
Plaque morphology analysis. **(A)** represents the agar plate images showing the plaques obtained after infecting *M. smegmatis* with either D29WT (WT; Left) or Δ*gp11* (right) phage. The diameter of the obtained plaques was measured using ImageJ software and plotted **(B)**. The experiments were performed at least three times and the total mean value of diameter (in mm) was plotted with standard deviation. *P* value; **< 0.05.

We next assessed morphology of the plaques obtained for D29Δ*gp11* phage and compared the same with the D29WT. *M. smegmatis* was infected with either D29Δ*gp11* or WT and the plaques were observed post 24 h incubation at 37°C. Interestingly, the plaques obtained for the D29Δ*gp11* are found to be significantly smaller than that obtained for the WT D29 phage (Figure 2A). We also measured the plaque size using ImageJ software (Schneider et al., 2012). The diameter of plaques for D29 WT and D29Δ*gp11* was found to be ∼5.5 and ∼4 mm, respectively (Figure 2B).

### D29Δ*gp11* Phage Shows Significantly Delayed Cell Lysis

**FIGURE 3:**
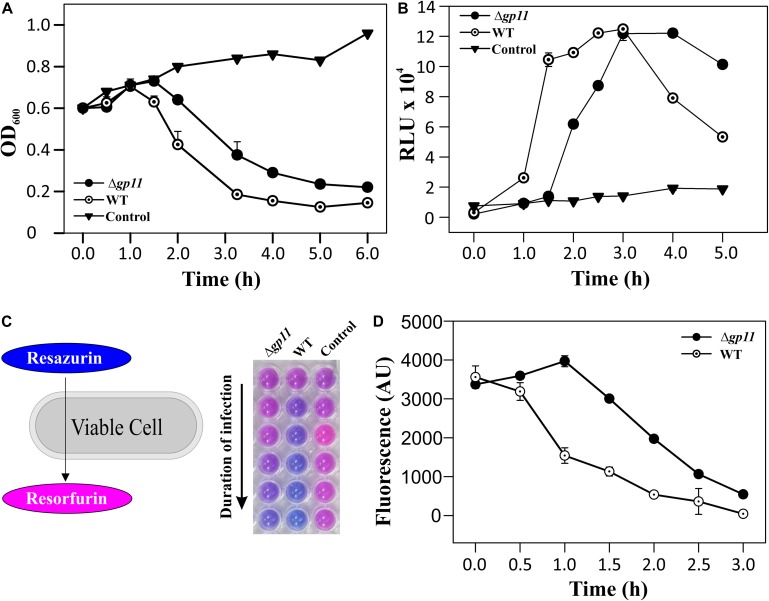
Phage infection with D29Δ*gp11* shows delayed host cell lysis. **(A)** depicts the lysis curve obtained after infecting *M. smegmatis* with either D29WT (WT) or D29Δ*gp11* (Δ*gp11*). The optical density of the culture at 600 nm (OD_600_) was measured at specified time points and plotted. **(B)** shows the ATP released into the culture supernatant at specified time points, in the form of relative luminescence unit (RLU). **(C)** shows the schematic for the alamarBlue assay; the blue color Resazurin dye is converted into pink colored Resorfurin by live cells. Here, a representative multiwell plate image is shown with *M. smegmatis* culture infected with phage with the duration of infection marked with an arrow; the change in color is observed due to presence of viable cells remaining after phage infection. In all the three panels, “Control” represents the *M. smegmatis* culture without D29 phage infection. In panel **(D)** fluorescence obtained from Resorfurin dye in the alamarBlue cell viability assay at specified time points is plotted for both D29WT (WT) and holin knockout (Δ*gp11*) phage-infected *M. smegmatis*. In panels **(A,B,D)**, only one representative plot is shown with error bars representing standard error.

In order to determine the effect of holin on phage-mediated cell lysis, we monitored the optical density of phage infected cultures. *M. smegmatis* log phase culture was infected with D29WT and D29Δ*gp11* at MOI = 1, and the OD_600_ was measured at regular intervals. Interestingly, whereas the culture infected with D29WT showed a decrease in OD_600_ at 90 min, the culture infected with D29Δ*gp11* phage demonstrated a significant delay in host cell lysis with an OD_600_ decreasing at 120 min (Figure 3A). The uninfected culture here was taken as negative control that showed normal growth. In order to further confirm the host cell lysis due to phage infection, we measured the ATP release upon cell lysis. The released ATP in the culture supernatant obtained for each time point samples was measured using the ATP estimation kit. Luminescence data thus obtained showed surge in ATP amounts in the culture supernatant at 90 min post-infection of *M. smegmatis* with D29WT phage, whereas no significant increase in the ATP level was detected in case of culture infected with D29Δ*gp11* at the same time point; in the latter, the ATP amount increased in the culture supernatant only after 120 min of infection (Figure 3B), clearly indicating a delay in the host cell lysis by the mutant phage. We also assessed the host cell viability during phage infection by following the alamarBlue assay (Warrier et al., 2012; Joshi et al., 2019; Patil and Jain, 2019). The assay was performed at different time points after infecting *M. smegmatis* with either D29WT or D29Δ*gp11* phage. Here, in presence of viable cells, the blue color dye is reduced to give fluorescent pink color (Figure 3C), which is then measured on a fluorescence spectrophotometer with an excitation/emission wavelengths as 540/590 nm, respectively. Expectedly, we observed that the cells infected with the D29Δ*gp11* phage were able to reduce the dye only at a significantly later time point as compared with the D29WT (Figure 3D). This data suggests that when *M. smegmatis* is infected with holin knockout phage, hosts cells are viable for longer time than when infected with WT phage.

### Mycobacteriophage D29 WT and Δ*gp11* Show Indifferent Morphology and Adsorption Kinetics

**FIGURE 4:**
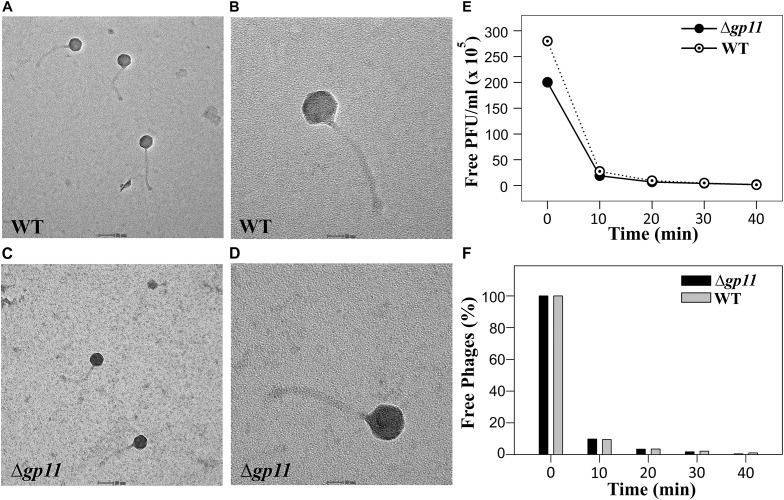
Transmission electron microscopy (TEM) and Phage adsorption kinetics. **(A–D)** show the TEM imaging of D29WT (Panels A & B) and D29Δ*gp11* **(C,D)**. The scale bar in **(A)** and **(C)** correspond to 50 nm; in **(B)** and **(D)**, it is 20 nm. **(E)** and **(F)** show the phage adsorption kinetics for both D29WT and D29Δ*gp11*, wherein a decrease in the free phage titre is plotted as free PFU/ml **(E)** and as percentage of free phages present in culture supernatant after infection **(F)**, with respect to time.

The delay in the host cell lysis observed in the case of D29Δ*gp11* phage tempted us to ask if it was because of differences in the morphology of the two phages or the attachment of phage to the host cells. We therefore first assessed the morphology of both the phages by transmission electron microscopy. Our data show that the two phages are identical in terms of general morphology (Figures 4A–D). We next performed adsorption kinetics with both the phages. Here, we allowed both WT and D29Δ*gp11* phages to attach to the log phase *M. smegmatis* culture at 37°C. This was followed by the determination of the fraction of free phages present in the supernatant at regular time interval by agar overlay method. The free phages obtained at different time point were plotted as free PFU/ml (Figure 4E) and also normalized with the free phages at initial time point (Figure 4F). Our data clearly show that nearly 90% of the phages (both WT and the mutant) are adsorbed within first 10 min of infection, thus suggesting that both WT and D29Δ*gp11* phages adsorb to host cell equally efficiently. We conclude from these data that the differences in the host cell lysis by the two phages are not contributed by either phage morphology or host attachment efficiency.

### D29Δ*gp11* Shows Increased Latent Period and Decreased Relative Phage Titre

**FIGURE 5:**
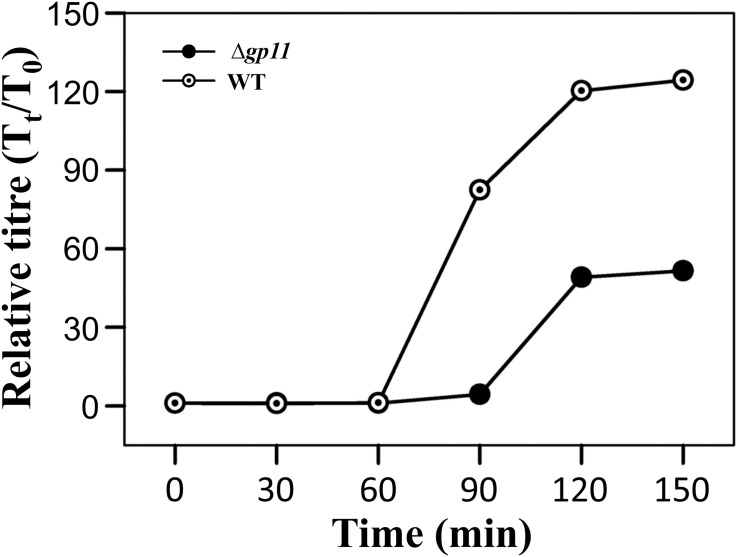
One step growth curve and latent period determination. The plots depict one step growth curve to determine the latent period of infection for both D29WT (WT) and D29Δ*gp11* (Δ*gp11*). The relative phage titre T_t_/T_0_, where T_0_ is the initial PFU and T_t_ is the PFU at time *t*, obtained at specified time points is plotted. Here, the latent period is the time after which the relative phage titre increases (for D29WT, it is 60 min, whereas for D29Δ*gp11*, it is 90 min).

Holin is known to be primarily involved in carrying out a timely lysis of the host bacterium. Conceivably, therefore, a deletion of holin would result in loss of this property of the phage and may affect phage physiology. We, therefore, carried out the one step growth curve analysis to examine the effect of holin deletion on the latent period of phage infection. Our data show that in the absence of holin, the latent period is drastically changed. We observe that when *M. smegmatis* log phase culture is infected with D29WT phage, there is a significant increase in the phage titre at 90 min. In contrast, such increase at 90 min is not observed when the culture is infected with D29Δ*gp11*; in this case, an increase in phage titre is seen only at 120 min. Our data suggest that the latent period of WT phage is ∼60 min, whereas it is 90 min for the D29Δ*gp11* phage (Figure 5). Thus an increase of 30 min in the latent period clearly indicates the importance of holin in the timing of lysis of the host bacterium at the end of lytic cycle after D29 phage infection. It also did not escape our notice that a rise in the relative phage titre of D29Δ*gp11* at later time point is ∼2 fold lower than that of the WT phage, which immediately suggests a decrease in the burst size of the D29Δ*gp11* phage. At this juncture, we believe that the less titre observed in D29Δ*gp11* phage is a result of an inefficient host cell lysis in the absence of holin, since the other two proteins, LysA and LysB, expressed efficiently in the cells infected with holin knockout phage. Taken together, our data suggest that holin is crucial for timely and efficient host cell lysis by D29 phage.

## Discussion

The underlying mechanism behind holin function is well understood in phages infecting *E. coli*. In addition to holin, several phages express antiholin, which prevents the premature hole formation. Mutations in these alleles have been shown to cause defective cell lysis, with holin null mutants giving rise to an absolute lysis defective phenomenon and phages losing their ability of plaque formation (Wang et al., 2000). In a separate study, point mutation in S^λ^ holin gene A52G has been shown to cause early lysis, thus reducing burst size as compared to WT allele (Johnson-Boaz et al., 1994).

Mycobacteriophage D29 is one of the few virulent phages that can infect and kill both slow-growing *M. tuberculosis* and fast-growing *M. smegmatis*. Host cell lysis by the D29 is solely timed by holin, and thus far, no antiholin has been discovered. In this manuscript, we show how holin protein is important for governing host cell lysis in a time-dependent fashion, and how a deletion of holin influences D29 physiology.

By generating holin knockout, we show that unlike *E. coli* phages, mycobacteriophage D29 is viable. However, we observed notably smaller plaques with the holin knockout phage. Further to check the “clock” function of holin, we measured the rate of host *M. smegmatis* cell lysis upon infection with knockout phage and compared that with D29WT. We find a remarkable difference in the timing of cell lysis exhibited by both the phages. Our experiments show that D29Δ*gp11* displays a 30 min delay in cell lysis as compared to the D29WT. This was further corroborated by the ATP release into the culture supernatant as a consequence of the cell lysis, and the cell viability assays carried out by measuring OD_600_ as well as AlamarBlue dye reduction. Moreover, this notable delay in the host cell lysis cannot be attributed to either a change in phage morphology or its adsorption property as confirmed by our TEM and phage adsorption kinetics data, respectively. Our electron microscopy data show that both WT and holin knockout phages have the same morphology. Additionally, the adsorption kinetics data suggest that nearly 90% of both WT and D29Δ*gp11* phages are adsorbed to the host cell within initial 10 min of phage addition, and no distinguishable differences could be observed. Finally, our one step growth curve experiment with the knockout phage shows an increase in the burst time and a decrease in the burst size. Here, the latent period was found to be increased by 30 min, which concurs well with all other data as discussed above. These observations are in agreement with a previous study with Ms6 phage, wherein a deletion of holin-like proteins such as *gp4* or *gp5* has been shown to cause early or delayed host cell lysis coupled with very small or large size plaques, respectively (Catalao et al., 2011). Taken together, our data show that while holin is important for the general maintenance of phage physiology, it is dispensable for phage viability. Thus the ability of D29Δ*gp11* to lyse host cell in the absence of holin with a simultaneous decrease in the burst size strongly indicates an inefficient host cell lysis.

Since canonical holins are involved in pore formation in the membrane to let the endolysin diffuse to the peptidoglycan (PG) layer (the substrate of endolysin), what allows D29 endolysin to function in a holin knockout phage? Previous works from our group and Payne & Hatfull have shown that the expression of D29 LysA in *M. smegmatis* results in cell lysis in a holin-independent manner (Payne and Hatfull, 2012; Pohane et al., 2014). These data strongly suggest that holin of D29 is not required for the LysA translocation to the periplasm. It is thus likely that LysA protein translocates to periplasm by secretion via a yet unidentified mechanism post-expression. However, the delay in cell lysis by D29Δ*gp11* phage can be explained by the “clock” function of holin. In other words, it is likely that while holin may not be required for LysA translocation, it is needed for the LysA to become fully active in the periplasm in a timely fashion, as has been suggested in the case of holin-independent (Young, 2002; Park et al., 2007; Briers et al., 2011) as well as canonical endolysins where membrane depolarization is necessary for their activation (Garcia et al., 2010; Proenca et al., 2015; Fernandes and Sao-Jose, 2016). For example, a previous work involving a canonical endolysin of *Bacillus subtilis* phage SPP1 suggested that holin is required for the activation of endolysin after its translocation to the periplasm (Fernandes and Sao-Jose, 2016). Nonetheless, whether D29 holin is involved in LysA activation (and probably its translocation also) is presently not known.

While these data may point to why holin deletion leads to a delay in the host cell lysis upon phage infection, they do not completely explain the observed lower burst size in the case of D29Δ*gp11*. For example, in an earlier study, we reported a D29 phage having a mutation in its LysA protein displayed a late lysis phenotype but with an increase in burst size (Joshi et al., 2019). However, a previous report on an esterase mutant (Δ*lysB*) of mycobacteriophage Giles suggested that LysB is required for efficient phage release (Payne et al., 2009). Similarly, deletion of LysB in Ms6 phage has also been shown to result in low number of phage progeny release after infection (Gigante et al., 2017). These studies allow us to suggest that in D29 phage, holin is required for the translocation of LysB. Taken together, we speculate that the lower burst size in D29Δ*gp11* is observed not only because LysB is unable to reach its site of action, i.e., mycolylarabinogalactan layer, but also because while LysA translocates to the periplasm in a holin-independent manner, it is not fully activated to perform its function in the absence of holin.

Phages have the capability to proliferate inside the host and lyse it. Hence, they are also known as “Self-replicating pharmaceuticals” (Payne et al., 2000). Mycobacteriophages are actively being considered for phage therapy against mycobacterial diseases including that caused by the pathogenic *M. tuberculosis* (Hatfull, 2014a) and other mycobacteria (Dedrick et al., 2019), due to the rapid emergence of drug resistant strains. We have earlier reported a mutant D29 phage with a late lysis phenotype but with an increase burst size. Since holin decides the “clock” function of the phage-mediated cell lysis, we believe that by incorporating altered holin gene in our holin knockout phage, novel mycobacteriophage with shorter latent period can be engineered, which may lead to a rapid clearance of bacteria from infection. Such designed phages can be readily used as novel next-generation phage-based therapeutics.

## Data Availability Statement

All datasets generated for this study are included in the article/Supplementary Material.

## Author Contributions

VJ conceived the idea. VB and VJ designed the research. VB performed the research. VB and VJ analyzed the data and wrote the manuscript.

## Conflict of Interest

The authors declare that the research was conducted in the absence of any commercial or financial relationships that could be construed as a potential conflict of interest.

## Supplementary Material

FIGURE S1 Recombineering of mycobacteriophage D29 using BRED (Bacteriophage Recombineering of Electroporated DNA) method for generating holin knockout. Panel A shows the schematic representation of the construction of AES (allelic exchange substrate). Mycobacteriophage D29 genomic DNA (gDNA) consists of lytic cassette encoding three overlapping genes *gp10*, *gp11*, and *gp12* shown as colored boxes. AES was constructed by performing overlapping PCR with the products generated from primers P1/P2 and P3/P4. Panel B shows agarose gel for the PCR amplified AES Up (Primer P1 and P2) and AES Down (Primer P3 and P4) fragments of ∼100 bp each. Panel C shows the AES of ∼200 bp generated by overlapping PCR using primers P1 and P4. In both panels, L represents the DNA ladder with two bands marked for their sizes. Panel D shows the schematic representation of recombineering between D29 gDNA and AES co-electroporated in *M. smegmatis* carrying pJV53 plasmid. pJV53 plasmid produces recombinase that allows for the homologous recombination between the D29 gDNA and AES.

FIGURE S2 Start and stop codons overlap for the lytic cassette genes. The DNA sequence on the top corresponds to the *gp10* and *gp11* gene sequences. The start codon (ATG) for *gp11* and the stop codon (TGA) for *gp10* are marked with arrows. Similarly, the DNA sequence at the bottom corresponds to the *gp11* and *gp12* gene sequences. The start codon (ATG) for *gp12* and the stop codon (TGA) for *gp11* are marked with arrows.

FIGURE S3 Screening of knockout phage by DADA PCR. Panel A shows the agar plate image containing plaques recovered after co-electroporation of phage gDNA and AES. A few of the plaques are marked with arrows. Panel B shows screening of individual recovered plaques (1–8) by DADA PCR (using primers P5 & P6). Panel C shows the schematic of the screening of plaques obtained after each phage infection of the positive samples obtained after performing DADA PCR in panel B. Panel D shows the desired amplification of ∼ 400 bp PCR product in all the pure knockout plaques (1–8) obtained after subsequent phage infections. In both B and D, L represents DNA ladder with few bands marked.

FIGURE S4 Sequence alignment of overlapping *gp10* and *gp12* after holin deletion in D29Δ*gp11*. Shown is the pairwise sequence alignment of the expected and the observed D29Δ*gp11* DNA. “Theoretical” sequence here corresponds to the DNA sequence that is expected after *gp11* deletion, whereas the “Sequencing” DNA depicts the DNA that was obtained after DNA sequencing. Blue and orange colored sequences represent *gp10* and *gp12*, respectively. Only a small region of the two genes is shown to depict the deletion of *gp11* from D29 genome, along with intact overlapping region as desired (underlined). ATG present in the underlined region is the start codon in *gp12*. “^∗^” represents consensus sequence.
