# Supplementary figures and images for: Deciphering the Role of Holin in Mycobacteriophage D29 Physiology
Source: Front Microbiol. 2020 May 8;11:883. doi: 10.3389/fmicb.2020.00883 (PMC7232613; doi:10.3389/fmicb.2020.00883)

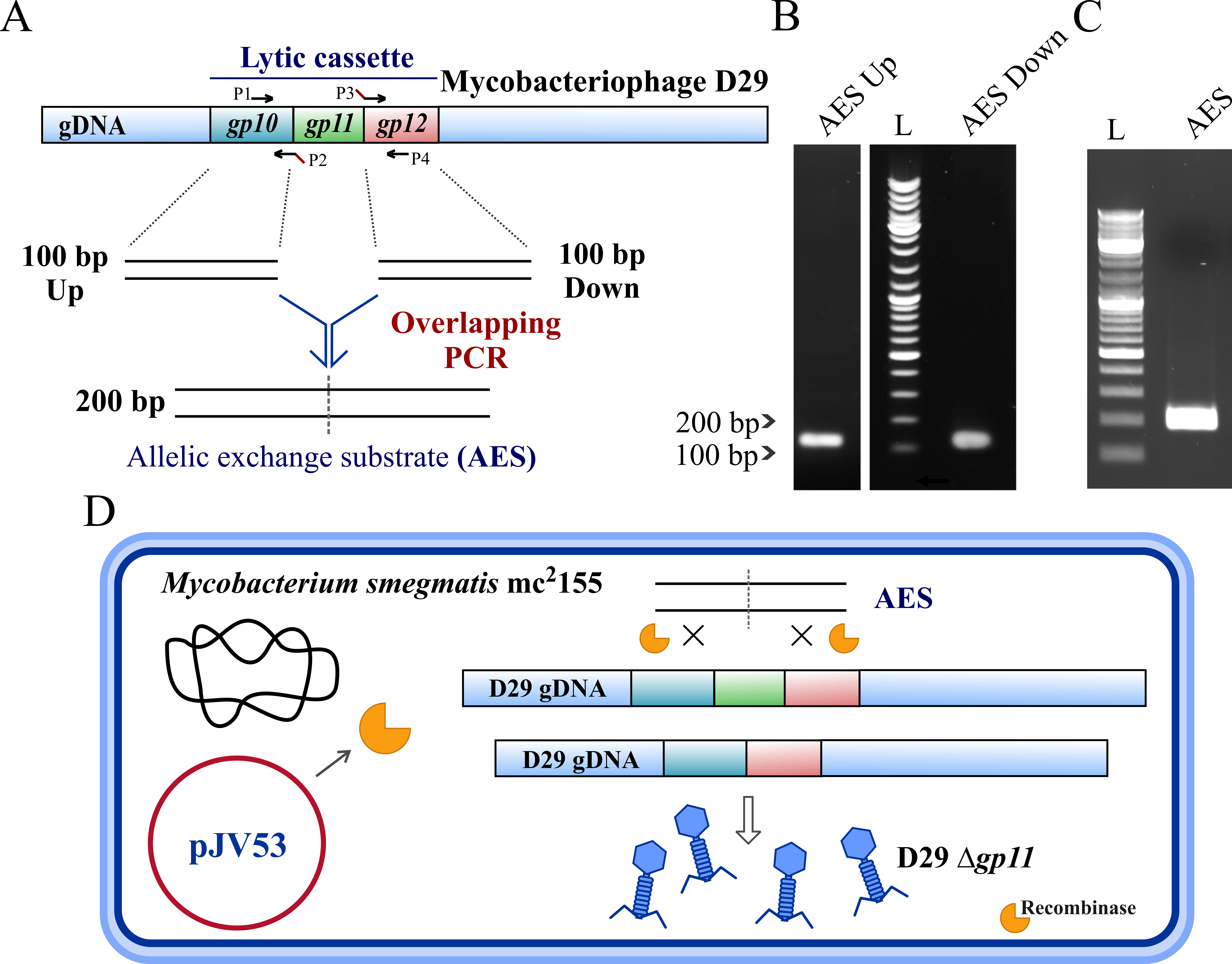

Supplement: FIGURE S1 — Recombineering of mycobacteriophage D29 using BRED (Bacteriophage Recombineering of Electroporated DNA) method for generating holin knockout. Panel A shows the schematic representation of the construction of AES (allelic exchange substrate). Mycobacteriophage D29 genomic DNA (gDNA) consists of lytic cassette encoding three overlapping genes gp10, gp11, and gp12 shown as colored boxes. AES was constructed by performing overlapping PCR with the products generated from primers P1/P2 and P3/P4. Panel B shows agarose gel for the PCR amplified AES Up (Primer P1 and P2) and AES Down (Primer P3 and P4) fragments of ∼100 bp each. Panel C shows the AES of ∼200 bp generated by overlapping PCR using primers P1 and P4. In both panels, L represents the DNA ladder with two bands marked for their sizes. Panel D shows the schematic representation of recombineering between D29 gDNA and AES co-electroporated in M. smegmatis carrying pJV53 plasmid. pJV53 plasmid produces recombinase that allows for the homologous recombination between the D29 gDNA and AES. [file Image_1.TIF]

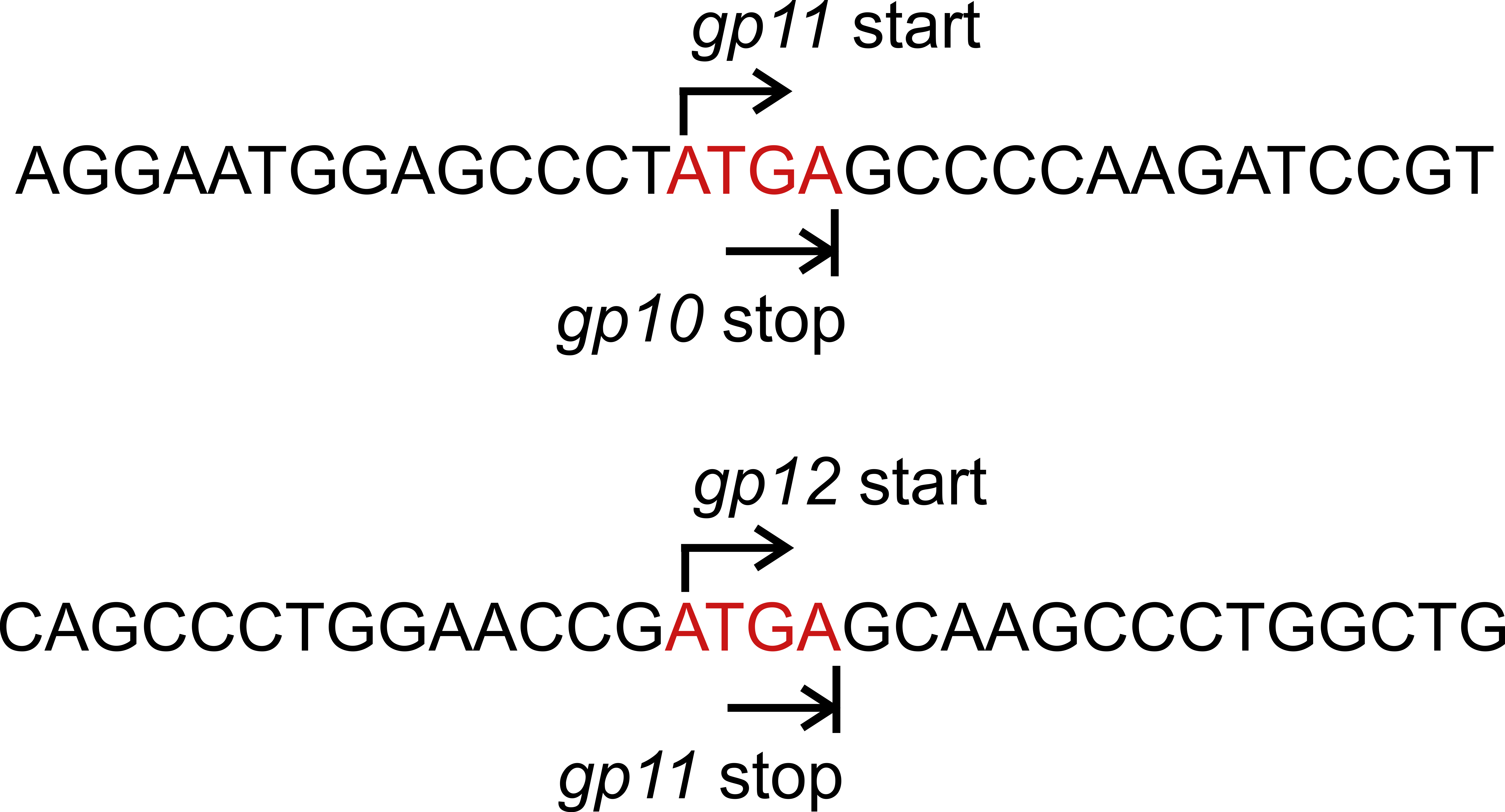

Supplement: FIGURE S2 — Start and stop codons overlap for the lytic cassette genes. The DNA sequence on the top corresponds to the gp10 and gp11 gene sequences. The start codon (ATG) for gp11 and the stop codon (TGA) for gp10 are marked with arrows. Similarly, the DNA sequence at the bottom corresponds to the gp11 and gp12 gene sequences. The start codon (ATG) for gp12 and the stop codon (TGA) for gp11 are marked with arrows. [file Image_2.TIF]

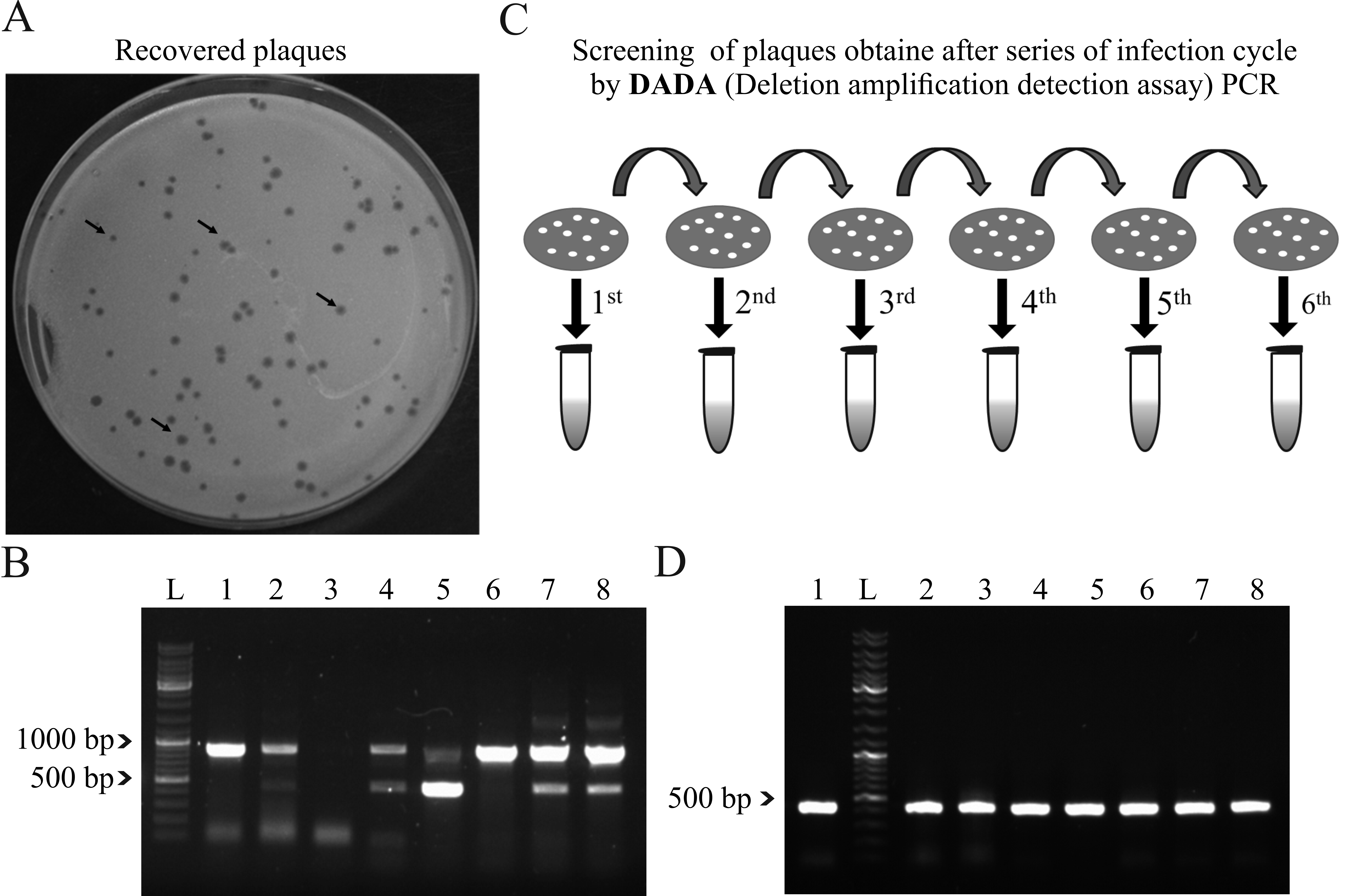

Supplement: FIGURE S3 — Screening of knockout phage by DADA PCR. Panel A shows the agar plate image containing plaques recovered after co-electroporation of phage gDNA and AES. A few of the plaques are marked with arrows. Panel B shows screening of individual recovered plaques (1–8) by DADA PCR (using primers P5 & P6). Panel C shows the schematic of the screening of plaques obtained after each phage infection of the positive samples obtained after performing DADA PCR in panel B. Panel D shows the desired amplification of ∼ 400 bp PCR product in all the pure knockout plaques (1–8) obtained after subsequent phage infections. In both B and D, L represents DNA ladder with few bands marked. [file Image_3.TIF]

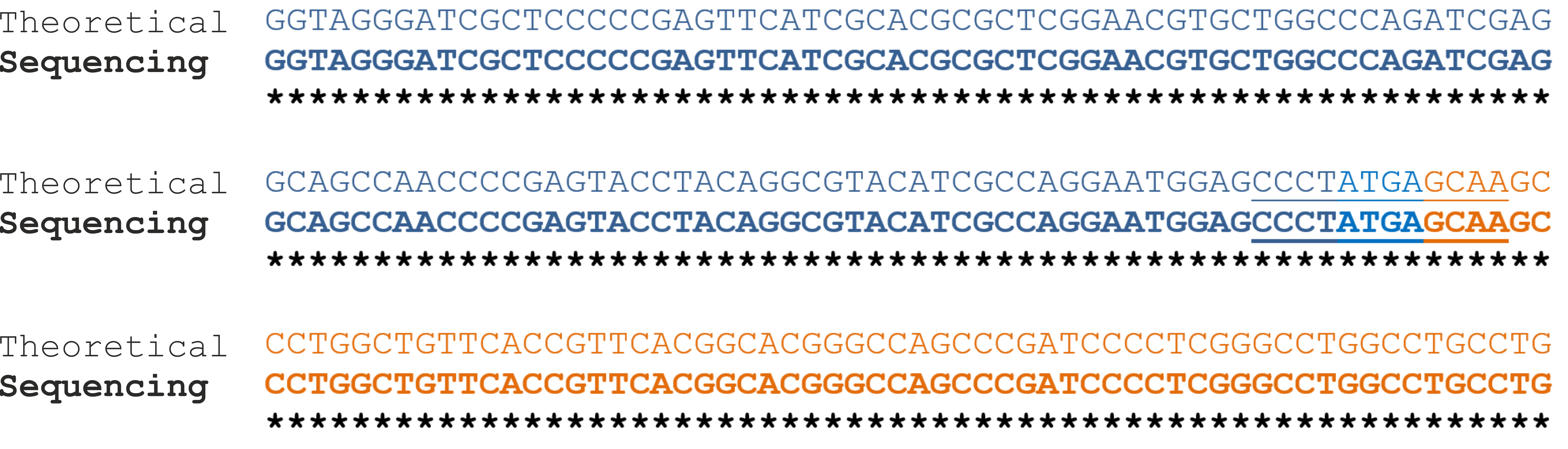

Supplement: FIGURE S4 — Sequence alignment of overlapping gp10 and gp12 after holin deletion in D29Δgp11. Shown is the pairwise sequence alignment of the expected and the observed D29Δgp11 DNA. “Theoretical” sequence here corresponds to the DNA sequence that is expected after gp11 deletion, whereas the “Sequencing” DNA depicts the DNA that was obtained after DNA sequencing. Blue and orange colored sequences represent gp10 and gp12, respectively. Only a small region of the two genes is shown to depict the deletion of gp11 from D29 genome, along with intact overlapping region as desired (underlined). ATG present in the underlined region is the start codon in gp12. “∗” represents consensus sequence. [file Image_4.TIF]
